# Common Problems Encountered During the Delivery of New Dentures Constructed by Dental Clinical Students at University Sains Islam Malaysia (USIM)

**DOI:** 10.7759/cureus.76003

**Published:** 2024-12-19

**Authors:** Nusima Mohamed, Sufi Athirah Shamsul Rahim, Fatihatun Najwa Mohd Nidzam, Nor Azlina Ismail, Aspalilah Alias

**Affiliations:** 1 Conservative Dentistry and Prosthodontics, Universiti Sains Islam Malaysia, Kuala Lumpur, MYS; 2 Dentistry, Universiti Sains Islam Malaysia, Kuala Lumpur, MYS; 3 Paediatric Dentistry and Orthodontics, Universiti Sains Islam Malaysia, Kuala Lumpur, MYS; 4 Anatomy, Universiti Sains Islam Malaysia, Kuala Lumpur, MYS

**Keywords:** acrylic denture, cobalt chromium, dental students, denture delivery, new denture, problems

## Abstract

Background

Dentures are one of the main treatments dentists deliver to restore the oral function and aesthetics of individuals with missing teeth. Clinical denture fabrication and manufacturing is one of the main training modules for undergraduates during dental clinical training. However, students often face problems during denture delivery due to multiple factors causing discomfort to patients, affecting the quality of the denture and the patient’s adaptation period to the denture. This study aims to study the relationship between common problems during the delivery of dentures constructed by third and fourth-year dental clinical students at University Sains Islam Malaysia as well as the causes and factors leading to faulty dentures.

Methodology

Clinical and denture examinations were done alongside two sets of validated questionnaires distributed to students and patients. Descriptive data were presented and analyzed using the chi-square test in SPSS Version 29 (IBM Corp., Armonk, NY, USA) with p-values <0.05 considered statistically significant to identify the relationship between common problems and their causes and factors.

Results

The most common problem during denture delivery was pain (47%) which was associated with mechanical factors (85%). Pain mostly occurred among cobalt chrome denture patients (60%). The longer the history of wearing dentures the more it contributed to patient psychological factors (p = 0.004). Although the type of denture was not associated with denture problems during delivery (p = 0.562), alveolar ridge resorption was likely associated with denture problems (p < 0.001).

Conclusions

This study demonstrated that the common problem encountered during denture delivery was related to faulty dentures instead of patient psychological problems.

## Introduction

Based on data from the National Oral Health Plan Malaysia 2011-2020, edentulism decreased in adults aged 15 to 54 years in 2000 from 53.9% in 1974 to 41.5% [1]. Partial or complete edentulism affects physical appearance, psychological and social relations, and mastication and speech [2]. Based on a study by Rodrigues et al., a denture is usually requested by patients to replace missing teeth to regain loss of function, aesthetics, and self-esteem [3]. However, delivering a removable prosthesis in the mouth contributes to changes that may negatively affect oral tissue. For this reason, a denture needs to be constructed correctly by a trained dentist.

Dental undergraduate students in Malaysia are expected to fulfill all requirements needed to graduate dental school and practice safely in the field of dentistry. These requirements help them gain exposure and be competent in their practice. In prosthodontics, students are required to construct both complete and partial dentures which includes every process of denture fabrication, both clinical and laboratory work, from taking impressions to denture processing and denture delivery. Each step is crucial in producing a non-faulty denture, ensuring a smooth denture delivery process. The University of Otago study demonstrated that undergraduate students can deliver high-quality complete dentures, as evidenced by 97% of patients experiencing minimal or no pain and 91% finding it easy to eat and swallow with their dentures. These results conclusively indicate that, under appropriate supervision, undergraduate dental programs can effectively train students to meet patient satisfaction standards in denture care [4].

On the other hand, the inability to deliver a good denture leads to complaints after delivery owing to patient discomfort or the inability to adapt due to many factors. According to a study from Malaysia, the main reasons for the repetition of the clinical steps during complete denture delivery were unfamiliarity with dental materials and the methods of handling them and lack of clinical skill [5]. This caused 36% of the students to require a mean of eight sessions for complete denture delivery which likely impacted their ability to complete requirements on time to graduate. This can also affect patient satisfaction and increase the review visit. The higher number of review visits can prolong the patient’s period of adaptability as well as lead to stressful clinical sessions for dental students. Hence, this study aims to identify the relationship between common problems encountered by undergraduate students during denture delivery and the causes of faulty dentures. This highlights the importance of thorough preparation and a strong understanding of patient management to ensure smooth denture delivery and successful follow-up visits.

## Materials and methods

This qualitative study evaluated patients treated with complete and removable partial dentures at Universiti Sains Islam Malaysia between April 2023 and October 2023. The patients were treated by third and fourth-year clinical dental students who were completing their prosthodontics requirements. These students had most first denture requirements delivered during the first year and had just completed their preclinical training on complete and partial dentures in the second year. To qualify for study inclusion, students needed to be involved in issuing their first set of dentures to patients aged 35-75 years, without any underlying medical conditions. The exclusion criteria excluded students who had issued more than one denture or had medical issues, particularly those outside the 35-75-year age range.

To determine the appropriate sample size for the study, a well-established statistical formula was employed that takes into account several key factors, such as population size, the desired confidence level, the hypothesized proportion of the population with the outcome, and the margin of error. For this calculation, a population size of 90 was used, representing the total number of potential participants comprising the total number of third and fourth-year students. A 95% confidence level was applied to ensure that the sample accurately reflected the population. Additionally, a margin of error of 5% was selected, which is a common standard in research, allowing for a small degree of error while maintaining reliable results. It was hypothesized that 50% of the population may exhibit the outcome being studied, as this is a conservative estimate often used when the true proportion is unknown.

Based on these parameters, the sample size calculation yielded a result of 74 participants. Ultimately, this calculated sample size provided a strong foundation for the research, ensuring that the results could be confidently generalized to the wider population while maintaining a manageable and feasible number of participants. Ethical approval was obtained from the ethical committee of Universiti Sains Islam Malaysia (approval number: USIM/JKEP/2023-252). All participants were given a thorough explanation of the study procedures and written informed consent was obtained.

The study consisted of the following two main parts: (a) the examination of the patients’ intraoral condition according to the standard clinical examination form (Appendix 1) and the constructed denture based on the standard denture examination form (Appendix 2) before the delivery and (b) validated questionnaire distribution to the student and their patient. The study assessed the problems encountered that may be affected by the mechanical aspect of the condition and quality of the delivered denture, the physiological aspect derived from the intraoral condition of the patients, and the psychological aspect related to the patient’s psychological condition which is their experience with denture wearing and adaptation. Part (a) aimed to assess the mechanical and physiological aspects, while part (b) mainly aimed to study the psychological aspects as well as the relationship between all the assessment aspects.

The examination of the intraoral and denture condition and quality was done by calibrated researchers and filled in the form presented in Appendix 1 and 2. The intraoral examination aimed to identify the relationship or exclude the oral tissue condition related to the difficulty of gaining good denture retention and stability. Severe resorption of the alveolar bone ridge or presentation of a flabby ridge as physiological aspects affect the mechanical aspect, such as retention of the denture, and psychological aspects, such as adaptability duration to the denture. The prosthesis examination included assessing the extension, polishing, and any denture defects such as porosity, crazing, or sharp edges to confirm that the denture meets the ideal condition of denture delivery. The level of porosity was measured as mild, moderate, and severe according to the coverage area of porosity. Porosity that needed rebase and was unacceptable to deliver was classified as severe. Moderate porosity referred to the coverage area of porosity not more than 50% and could be repaired. Mild porosity was classified as spot porosity mainly due to gaseous porosity that could be removed, repaired, and polished during denture polishing and finishing.

The questionnaire was validated according to a systematic four-phase data collection process. The first phase involved translating the questionnaire into multiple languages to ensure accessibility and inclusivity. The second phase focused on content validation, where the questionnaire was tested and pre-tested among 10 individuals to assess its clarity, relevance, and comprehensiveness. Phase three entailed validation by a specialist prosthodontist, ensuring that the questionnaire’s content aligns with professional standards and expertise. Finally, phase four involved distributing the validated questionnaire to the participants, marking the commencement of data collection. This multi-step validation process aimed to ensure the questionnaire’s quality, reliability, and suitability for collecting accurate and meaningful data.

The student’s questionnaire (Appendix 3 and Appendix 4) consisted of three sections, i.e., demographic data, factors associated with denture problems, and student management to overcome the problems. The questionnaires administered to students aimed to analyze the relationship between denture delivery problems and mechanical and physiological aspects. The questionnaire for patients (Appendix 5 and Appendix 6) was adapted from the studies by Ahmad et al. and Namano et al. which consists of comfort, satisfaction, and perception toward function and aesthetics [6,7]. This questionnaire was distributed to identify the psychological aspects as well as to assess the relationship between psychological aspects with problems encountered during denture delivery.

Data were analyzed using SPSS version 29 (IBM Corp., Armonk, NY, USA). Categorical variables are presented as frequency and percentages of data. Demographic variables and factors related to denture problems were analyzed using the chi-square test. P-values <0.05 were considered statistically significant.

## Results

The study involved 74 clinical dental students as participants. The type of denture issued included full acrylic dentures at 36 (49%), partial cobalt-chrome dentures at 25 (34%), and partial acrylic dentures at 13 (17%). The most common complaint received upon denture delivery was pain (35, 47%), followed by bulky (22, 30%), loose (13, 18%), and difficulty swallowing/gagging (4, 5%). Factors associated with faulty dentures were mainly mechanical (63, 85%), followed by psychological (7, 10%) and physiological (4, 5%).

**Figure 1:**
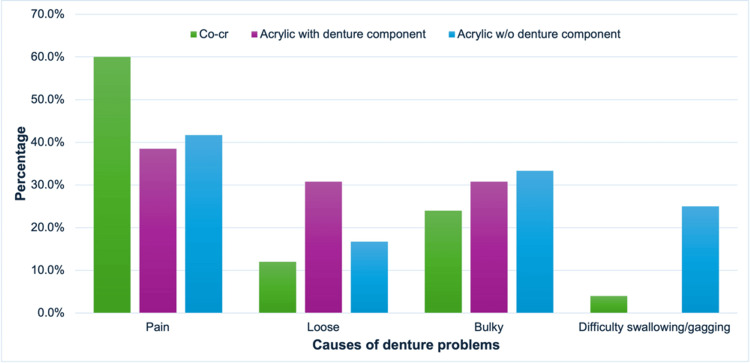
Percentage of denture problems and the causes of the problems. Co-Cr: cobalt-chrome

A descriptive analysis of the relationship between the type of problems during the delivery visit and its causes is presented in Figure 1. Mechanical factors were the most common contributors to all types of denture problems upon denture delivery.

**Figure 2:**
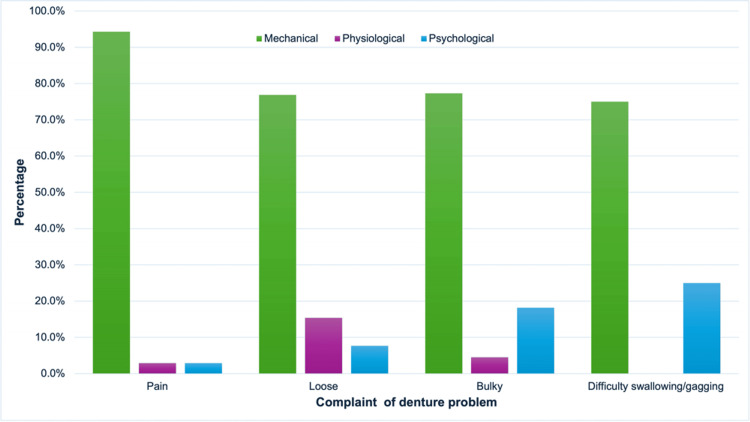
Problems encountered during denture delivery and the type of denture.

For the problems encountered during denture delivery in different types of dentures, partial cobalt-chrome dentures showed the least problems while acrylic partial dentures were associated with the most problems. Pain was the largest problem for partial cobalt-chrome (44, 60.0%) and partial acrylic (28, 38.5%) dentures (Figure 2). Chi-square analysis showed no association between the type of denture and problems faced during the delivery visit (p = 0.562).

**Figure 3:**
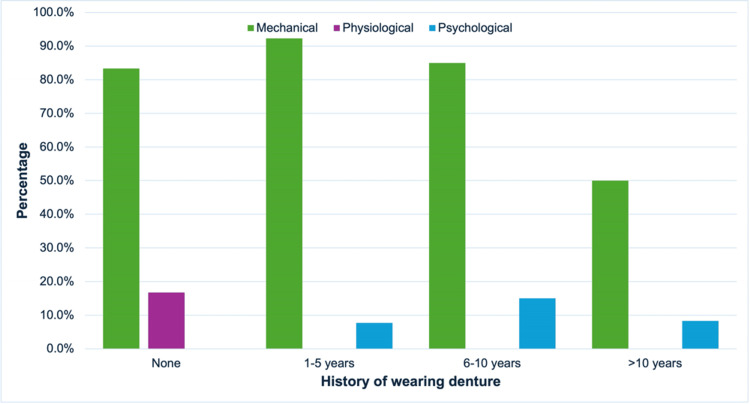
Relationship between the history of wearing dentures and denture problems.

The history of wearing dentures was also associated with denture problems during the delivery visit. For patients who did not have a history of wearing dentures or had a history of wearing dentures of 1-10 years, the most contributing factor was the mechanical factor at 61 (83.3%) to 68 (92.3%) (Figure 3). In patients with more than 10 years of denture history, denture problems likely occurred due to mechanical or psychological factors. Mechanical factors encompassed the physical aspects of the denture and its interaction with the oral environment, including wear and tear, changes in oral tissues, loose or broken attachments, poor oral hygiene, and improper denture care. Conversely, psychological factors included patients’ mental state and how it influenced their denture experience, including self-consciousness, frustration, body image issues, and even depression stemming from denture-related discomfort and limitations. A significant association was found between a history of wearing dentures and denture problems (χ²(6) = 19.035, p = 0.004) (Table 1).

**Table 1:** The chi-square test findings of the relationship between a history of wearing dentures and denture problems. ^a^: Nine cells (75.0%) have an expected count of less than 5. The minimum expected count is 0.22. ^b^: Computed only for PxP table, where P must be greater than 1.

	Value	df	Asymptotic significance (two-sided)
Pearson chi-square	19.035^a^	4	0.004
Likelihood ratio	18.451	4	0.005
Linear-by-linear association	3.602	1	0.058
McNemar-Bowker test	-	-	b
Number of valid cases	74		

**Figure 4:**
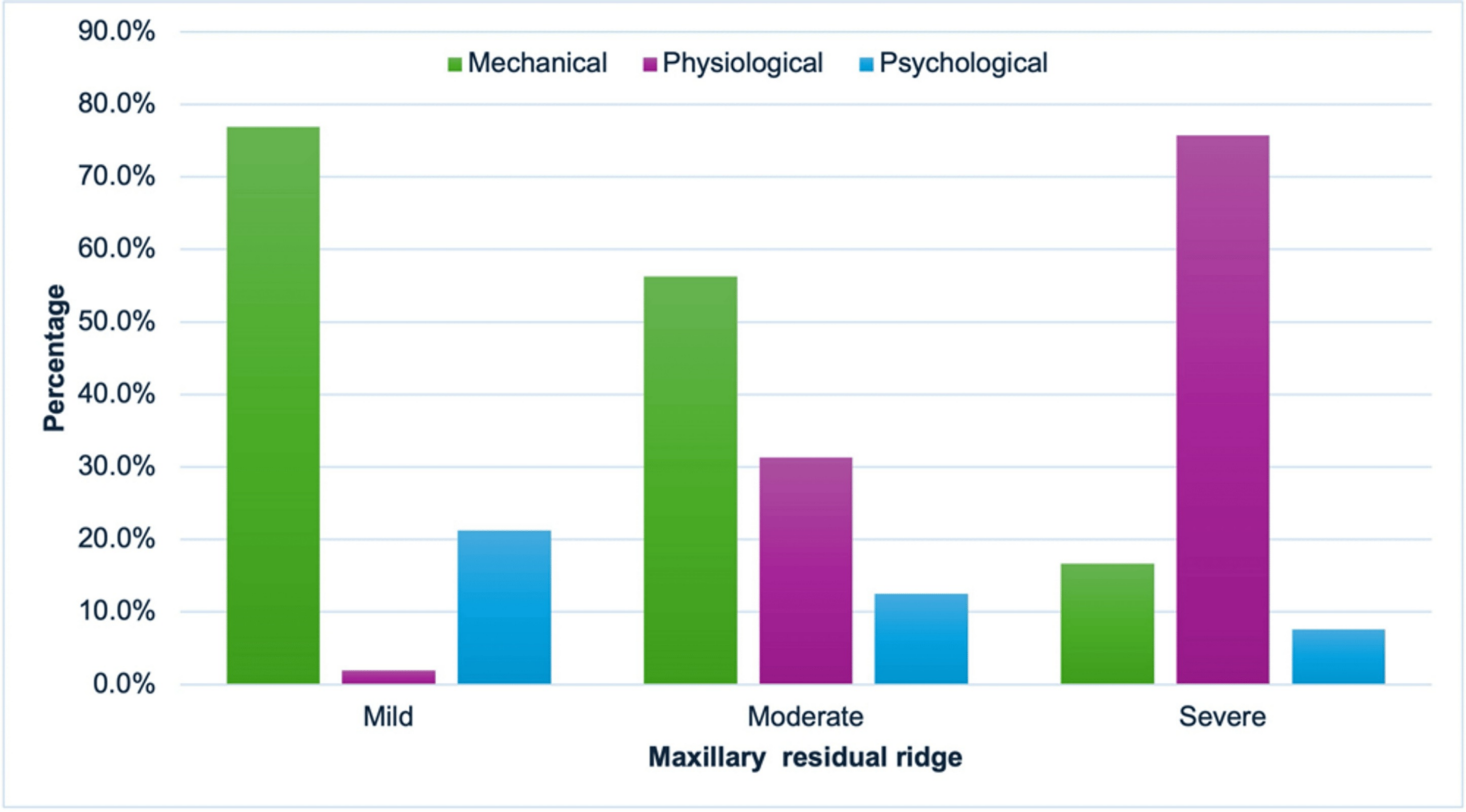
Relationship between maxillary ridge resorption and denture problems.

**Figure 5:**
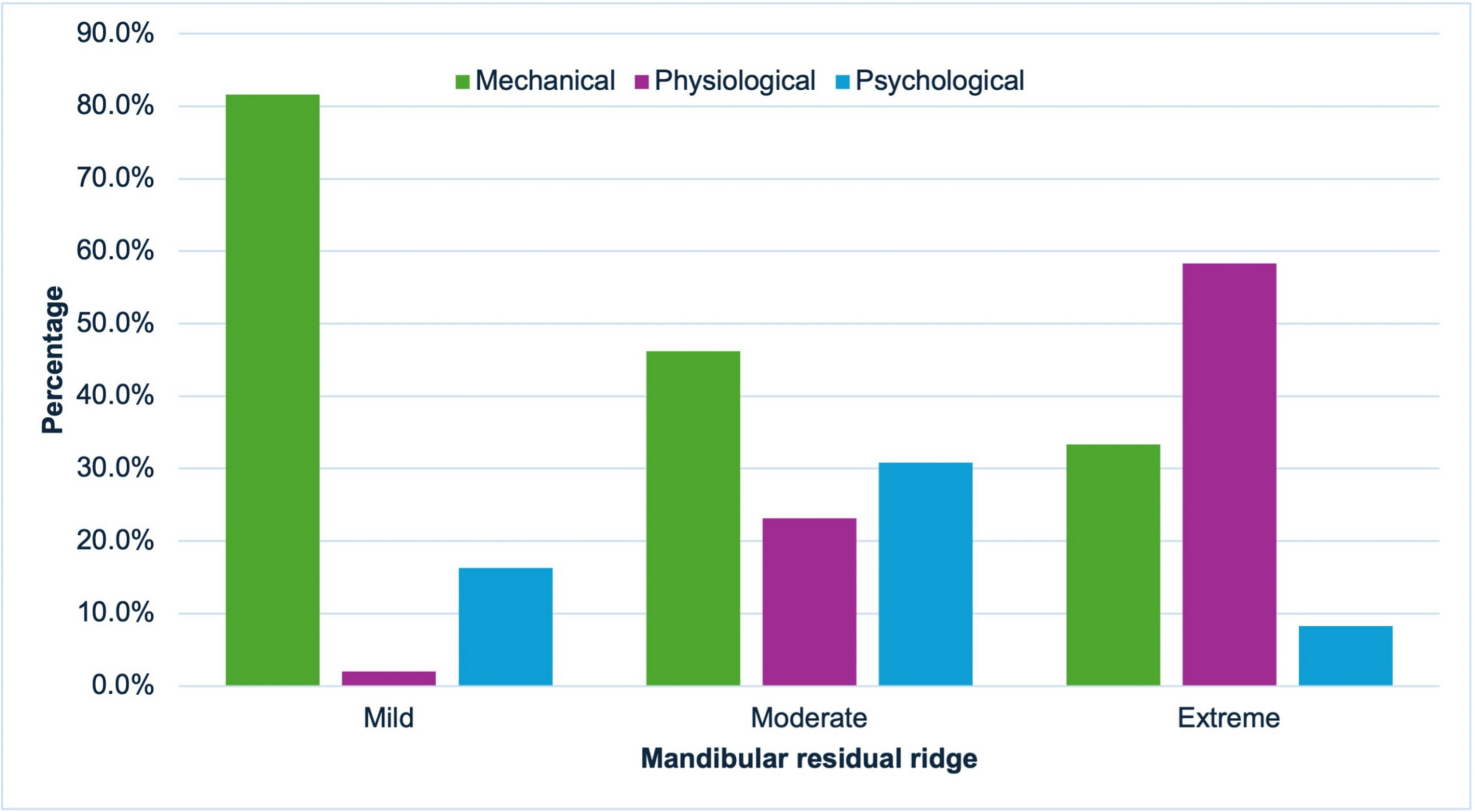
Relationship between mandibular ridge resorption and denture problems.

The relationship between ridge resorption and denture problems was evaluated. In maxillary ridge resorption, the more severe the resorption, the more problems occur due to physiological factors and reduced mechanical and psychological factors (Figure 4). In mandibular ridge resorption, the more severe the resorption, the higher the percentage of problems in physiological factors and reducing problems in mechanical factors. These findings indicate that physiological factors contribute more significantly to maxillary ridge resorption than to mandibular ridge resorption (Figure 5). A significant association was found between residual ridge resorption and denture problems (χ²(df) = 25.16, p < 0.001) (Table 2).

**Table 2:** The chi-square test findings of the relationship between residual ridge resorption and denture problems. ^a^: Five cells (55.6%) have an expected count of less than 5. The minimum expected count is 0.89.

	Value	df	Asymptotic significance (two-sided)
Pearson chi-square	25.160^a^	4	<0.001
Likelihood ratio	17.885	4	0.001
Linear-by-linear association	1.193	1	0.275
McNemar-Bowker test	10.347	3	0.015
Number of valid cases	74		

## Discussion

This study showed that the most common complaint during denture delivery is pain on insertion of the denture, followed by bulky, loose, and difficulty swallowing or gagging. These problems are usually associated with an overextended denture flange, sharp edges of the denture fitting surface, incorrect placement of the clasp causing impingement on the soft tissue, and incorrect occlusal registration. These findings are comparable to a study from India by Sharma et al. which reported that the most common complaint during denture delivery was discomfort or pain [8]. These denture problems can be due to several factors, such as the fitting of the dentures (mechanical), the patient’s oral condition (physiological factors), or the patient’s adaptation to the new appliance (psychological factors).

Patients usually need a review session for any adjustments after a week of denture delivery. However, in a previous study, in 63 (85.5%) patients, dentures needed earlier adjustment within 24 hours after insertion; however, the percentage reduced for further adjustment after the first adjustment was done [9]. A study by Kivovics et al. showed that denture care for the patient does not end after delivery but needs further review while the patient is in the adaptation period. By making necessary adjustments at the delivery stage and during follow-up appointments, clinicians can minimize the risk of denture irritation and ensure a more comfortable experience for the patient. Most denture adjustments are done by trimming the denture border at the retromylohyoid area, retromolar pad, and buccal sulcus [10]. As this adjustment is commonly done, dental students need to have a thorough understanding of adequate denture extension and how to gain a good denture-bearing area during border molding and secondary impression taking [11].

In this study, approximately 46 (62.9%) students managed their patients’ complaints properly, even though it was likely challenging for them. This shows that this research must seek to expand dental students’ understanding of the common problems that may occur during denture delivery so that they are aware of potential problems. Thus, they can be proactive in preventing or addressing these problems when they occur. This leads to better patient care and a more positive patient experience.

In this study, patients reported more complaints of pain and discomfort toward cobalt-chrome and acrylic partial dentures (34, 46.7%). This finding is supported by previous studies that concluded higher patient complaints among partial denture patients compared to complete dentures who also have lower quality of life ratings [12,13]. Furthermore, Bae et al. reported that partial denture patients have more problems with functional limitations (B = 0.200, p = 0.080), physical pain (B = 0.215, p = 0.086), and psychological discomfort (B = 0.207, p = 0.085) than complete denture that aligns with our study [12].

Studies have reported that one of the important factors in patients who have trouble adapting to new dentures is psychological factors (p < 0.001) [14-16]. This may be due to the strong relationship between satisfaction with the existing denture and the experience of long-term denture wearing [17]. This is also proven in this study showing a significant relationship between denture-wearing history with the problem or dissatisfaction, most probably due to the patient’s psychological state, of the new denture. As the duration of wearing increased, patient complaints during post-insertion increased. This is supported by a study that showed evidence of a negative correlation between age and comfort of use expectation (p = 0.0041, -44.41%). Hence, the longer the edentulism period, the higher the expectation level of the patients to the new dentures, with the hope that the denture will fit and function equally to or even better than their natural teeth [16].

Due to their high expectation, the treatment demand of such patients also increases [18]. Thus, this study showed that the experience of wearing a denture and the duration of wearing the existing denture would be a valuable prognostic factor influencing the outcome of the successful treatment of a patient with conventional dentures. Therefore, dental students need to learn to improve their communication in explaining the possible outcome of the dentures as well as gain patients’ trust in the clinical procedure of denture construction by students.

The mandibular arch had a higher rate of resorption than the maxillary arch, which is supported by a study by Raj et al. that reported that resorption occurs at a faster rate in the mandibular arch, with 60% of the patients having mandibular height reductions compared to the maxillary arch [19]. This is due to the area of the maxilla being generally larger than the mandible, which can distribute the forces of mastication more evenly, leading to less resorption [19]. Moreover, there is a variation in the quality of bone between the two jaws. The bone in the mandible is denser and more brittle than the maxilla, making it more prone to resorption. However, the progressive and irreversible mandibular alveolar resorption rate at an early stage of edentulism slows down with bone loss, longevity of edentulism, and attendant wearing of dentures [20]. By delivering a good fitting and comfortable denture the progress of bone resorption may be slow and stable. Understanding the oral anatomy of the correct denture-bearing area is crucial for dental students. This increases patient satisfaction and reduces dental student stress and anxiety due to unexpected outcomes of the denture construction.

One of the main limitations of this study is the relatively small sample size, with a total of 74 clinical dental students participating, and the study being conducted at a single institution (Universiti Sains Islam Malaysia). This may limit the generalizability of the findings to other dental schools or a broader population of dental students and patients. Furthermore, the study only included patients without medical illnesses, which may exclude a significant proportion of the elderly population who are often more likely to require dentures and may present with complex medical conditions that could influence denture success. Additionally, the study focused primarily on mechanical issues associated with dentures, such as pain and discomfort, while psychological and physiological factors, although considered, were not as extensively examined. Therefore, other important factors contributing to patient dissatisfaction, such as anxiety or systemic health conditions, may not have been fully explored. The reliance on self-reported data from both students and patients likely introduced potential bias, as respondents may have underreported or misremembered their experiences. Finally, the short duration of the study (April 2023 to October 2023) may not have allowed for the long-term follow-up necessary to assess issues such as the durability of dentures and long-term patient adaptation, further limiting the scope of the findings.

## Conclusions

The study showed that a significant number of patients experience discomfort or pain during the denture delivery visit. Most patient complaints can be attributed to mechanical factors, indicating a need for the improvement of denture fitting and adjustment procedures. However, it is also important to note the role of psychological factors in patient discomfort, suggesting that patient education on denture care and treatment as well as psychological support may be beneficial. Furthermore, physiological factors also contribute to the overall patient experience, indicating a need for individualized treatment plans that consider the unique physiological characteristics of each patient.

## Appendices

Appendix 1: Standard form of clinical examination

**Table 3:** Standard form of clinical examination.

Clinical examination form				
1.	Mucosa		Normal	Abnormal, specify:
2.	Saliva		Normal	Abnormal, specify:
3.	Maxillary torus		No	Yes, specify:
4.	Mandibular torus		No	Yes, specify:
5.	Depth of palatal vault		1. Shallow; 2. Deep; 3. Average	
6.	Maxillary residual ridge	Resorption	1. Mild; 2. Moderate; 3. Extreme	
		Contour/Shape	1. Regular; 2. Irregular, specify:	
		Quality of mucosa	1. Firm; 2. Flabby	
		Thickness of mucosa	1. Thin; 2. Normal; 3. Thick	
		Shape of the arch	1. U-shaped; 2. V-shaped	
7.	Mandibular residual ridge	Resorption	1. Mild; 2. Moderate; 3. Extreme	
		Contour/Shape	1. Regular; 2. Irregular, specify:	
		Quality of mucosa	1. Firm; 2. Flabby	
		Thickness of mucosa	1. Thin; 2. Normal; 3. Thick	
		Shape of the arch	1. U-shaped; 2. V-shaped	
8.	Maxillary peripheral tissue attachment	Labial frenum	1. Normal; 2. Low	
		Buccal frenum	1. Normal; 2. Low	
9.	Mandibular peripheral tissue attachment	Labial frenum	1. Normal; 2. Low; 3. High	
		Buccal frenum	1. Normal; 2. Low; 3. High	
		Lingual frenum	1. Normal; 2. Low; 3. High	
10.	Oral hygiene		1. Good; 2. Fair; 3. Poor	

Appendix 2: Standard form of denture examination

**Table 4:** Standard form of denture examination.

Criteria	Maxillary denture	Mandibular denture
Aesthetic	1. Good; 2. Fair; 3. Poor	1. Good; 2. Fair; 3. Poor
Retention	1. Good; 2. Fair; 3. Poor	1. Good; 2. Fair; 3. Poor
Stability	1. Good; 2. Fair; 3. Poor	1. Good; 2. Fair; 3. Poor
Extension	1. Good; 2. Fair; 3. Poor	1. Good; 2. Fair; 3. Poor
Occlusion	1. Good; 2. Fair; 3. Poor	1. Good; 2. Fair; 3. Poor
Porosity	1. Mild; 2. Moderate; 3. Severe	1. Mild; 2. Moderate; 3. Severe

Appendix 3: Student denture assessment form (Section A)

**Table 5:** Student denture assessment form (Section A).

Student background		
1.	Name	
2.	Year	
3.	Type of denture issued	□ Cobalt-chromium
		□ Acrylic with denture components
		□ Acrylic without denture components

Appendix 4: Student denture assessment form (Section B)

**Table 6:** Student denture assessment form (Section B).

Causes of the faulty denture		
1.	Does the patient complain about the new denture during denture delivery?	□ Yes, state the complaints (if multiple, state all):
		□ No (go to question 3)
2.	What is the cause of patient complaint?	□ Mechanical, state the problems:
		□ Physiological, state the problems:
		□ Psychological, state the problems:
3.	Do you encounter any faulty of the denture before the delivery visit?	□ Yes, state the faulty (if multiple, state all):
		□ No
4.	Which technique do you use to overcome the problems?	□ Trim denture. Specify which area:
		□ Extend denture. Specify which area:
		□ Selective grinding
		□ Re-register bite
		□ Reline/Rebase
		□ Redo denture
		□ Repair denture
		□ Add/Remove component
5.	Are you able to issue the denture in one visit?	□ Yes
		□ No, state the problems:

Appendix 5: Patient denture assessment form (Section A: patient background)

**Table 7:** Patient denture assessment form (Section A: patient background).

Patient background		
1.	Name	
2.	Age	
3.	Gender	□ Male □ Female
4.	Do you have a history of wearing dentures previously?	□ Yes □ No If yes, what is the age of existing dentures?
5.	Period of edentulism	□ 1–5 years □ 5–10 years □ More than 10 years

Appendix 6: Patient denture assessment form (Section B: patient assessment on comfortability, perceptions, and satisfaction)

**Table 8:** Patient denture assessment form (Section B: patient assessment on comfortability, perceptions, and satisfaction).

No.	Question	Yes	No
1.	Do you have any areas painful to pressure?		
2.	Do you feel pain on insertion and removal of the dentures?		
3.	Do you have difficulty swallowing?		
4.	Do you have pain at the periphery of the dentures?		
5.	Do you feel any burning sensation?		
6.	Do you feel any looseness of the denture?		
7.	Do you feel the tilting or rocking of the denture?		
8.	Do you have errors during occlusion?		
9.	Do you have a gagging reflex?		
10.	Does the lower denture rise when the mouth is opened?		
11.	Does the lower denture rise when the tongue is protruded?		
12.	Are you satisfied with the denture’s appearance?		
13.	Are you happy with the teeth’s appearance and color?		
